# Stomach-Specific Drug Delivery of Clarithromycin Using a Semi Interpenetrating Polymeric Network Hydrogel Made of Montmorillonite and Chitosan: Synthesis, Characterization and In Vitro Drug Release Study

**DOI:** 10.15171/apb.2019.019

**Published:** 2019-02-21

**Authors:** Yunes Panahi, Afshin Gharekhani, Hamed Hamishehkar, Parvin Zakeri-Milani, Hamed Gharekhani

**Affiliations:** ^1^Chemical Injuries Research Center, Systems Biology and Poisonings Institute, Baqiyatallah University of Medical Sciences, P.O. Box 1435916-471, Tehran, Iran.; ^2^Drug Applied Research Center, Department of Clinical Pharmacy (Pharmacotherapy), Tabriz University of Medical Sciences, Tabriz, Iran.; ^3^Drug Applied Research Center, Tabriz University of Medical Sciences, Tabriz, Iran.; ^4^Drug Applied Research Center and Faculty of Pharmacy, Tabriz University of Medical Sciences, Tabriz, Iran.

**Keywords:** Semi-interpenetrating polymer network, Chitosan, Montmorillonite, Clarithromycin, Gastroretentive drug delivery formulation

## Abstract

***Purpose:*** In this study, we aimed to prepare an extended drug delivery formulation of clarithromycin (CAM) based on a semi-interpenetrating polymer network (semi-IPN) hydrogel.

***Methods:*** Synthesis of semi-IPN hydrogel nanocomposite made of chitosan (CS), acrylic acid (AA), acrylamide (AAm), polyvinylpyrrolidone (PVP), and montmorillonite (MMT) was performed by free radical graft copolymerization method. Swelling kinetic studies were done in acidic buffer solutions of hydrochloric acid (pH = 1.2), acetate (pH = 4), and also distilled water. Also, the effects of MMT on the swelling kinetic, thermal stability, and mechanical strength of the hydrogels were evaluated. Moreover, in vitro release behavior of CAM and its release kinetics from hydrogels were studied in a hydrochloric acid buffer solution.

***Results:*** Fourier transform infrared spectroscopy (FTIR) results revealed that synthesis of semi- IPN superabsorbent nanocomposite and CAM incorporation into hydrogel was performed, successfully. Introducing MMT into hydrogel network not only improved its thermal stability but also increased mechanical strength of the final hydrogel product. Also, in comparison with neat hydrogel (1270 g/g), hydrogel nanocomposite containing 13 wt% MMT exhibited greater equilibrium swelling capacity (1568 g/g) with lower swelling rate. In vitro drug release experiments showed that CS-g-poly(AA-co-AAm)/PVP/MMT/CAM formulation possesses a sustained release character over extended period of time compared with CS-g-poly(AA-co- AAm)/PVP/CAM formulation.

***Conclusion:*** In the presence of MMT, the effective life time of drug is prolonged, demonstrating a sustained release property. The reason is that interlinked porous channels within superabsorbent nanocomposite network hinder penetration of aqueous solutions into hydrogel and subsequently cause a slower drug release.

## Introduction

Among different routes used for pharmacotherapy of diseases, oral administration has attained great attention due to the various merits such as patient satisfaction, low‐cost periodontal therapy, and flexibility in manufacturing technology and formulation strategy.^1,2^ By considering favorable compatibility with drugs and sustained release behavior over a prolonged period of time, utilization of superabsorbent hydrogels to prepare oral dosage forms of medications has recently emerged as an effective technique for treatment of stomach diseases.^3,4^ Superabsorbent hydrogels are polymeric materials with three-dimensional crosslinked network, which can absorb and preserve higher amounts of aqueous solutions within own structure without dissolving. Their exceptional features have tailored them for use in various fields such as tissue engineering, soft contact lenses, wound dressing, bioseparation, and drug delivery systems.^5-8^ To gain a more expanded hydrogel network in acidic medium of stomach, grafting of acrylate-based monomers onto polycationic backbone of a natural polysaccharide such as chitosan (CS) is indispensable.^9^ CS, a weak polybase biopolymer, consists of D-glucosamine and N-acetyl-D-glucosamine units, which is derived from N-deacetylation of chitin.^8^ The large quantity of reactive amino groups on CS backbone as well as its good mucoadhesion property enables it to be applied as good pH-responsive polymer in composition of stomach-targeted drug delivery formulations.^10,11^

*Helicobacter pylori* is a gram negative, spiral, microaerophilic, and multi-flagellate bacterium that lives both within and beneath of the adherent mucus layer of the stomach.^12,13^ The enzymes and toxins released by this bacterium may injure the gastric epithelial cells, resulting in diseases such as gastritis, gastroduodenal ulcer, and also gastric cancer.^14,15^ Therefore, eradication of *H. pylori* by antibiotics such as amoxicillin, clarithromycin (CAM), tetracycline, and metronidazole along with gastric acid suppressant is prerequisite for curing *H. pylori* related gastric diseases.^16^

CAM, due to the good stability in acidic medium of stomach and also short biological half-life of 3-5 hours, has been widely used to treat *H. pylori* infection.^17,18^ In order to achieve a higher efficiency of eradication, the effective concentration of antibiotic should be retained over an extended period of time within stomach through prolonging residence time. This purpose can be accomplished by increasing swelling capacity and also improving mechanical strength of the prepared oral dosage forms.^12^ In this regard, compounding of synthetic polymers with natural polysaccharides as semi-interpenetrating polymer network (semi-IPN) can be most beneficial.

Semi-IPN hydrogels are composed of crosslinked polymeric network in which two polymers have finely blended together without any covalent bonds between them and one polymer has interpenetrated through crosslinked network of other.^19^ Polyvinylpyrrolidone (PVP) is a water-soluble, non-toxic, biocompatible, and biodegradable synthetic polymer, which due to the good compatibility with other materials, it can be combined well with other polymers as IPN or semi-IPN hydrogels.^20^

In order to alleviate final production cost and also amend swelling capacity and mechanical characteristics of a semi-IPN hydrogel, introducing low cost clay minerals can be most beneficial. Among inorganic clay minerals, montmorillonite (MMT), due to the small particle size, stiffness, high in-plane strength, intercalation feature, and high aspect ratio, has been extensively used as filler in the composition of the gastroretentive drug delivery systems.^21^

In this study, chitosan-g-poly(acrylic acid-co-acrylamide)/PVP/MMT (CS-g-poly(AA-co-AAm)PVP/MMT) semi-IPN superabsorbent nanocomposite was first synthesized by free radical graft copolymerization method. Then, CAM was loaded in the hydrogel network. The effect of MMT on the swelling behavior, thermal stability, and also mechanical strength of the hydrogels was studied. The in vitro drug release behavior as well as drug release kinetics of the prepared formulations was evaluated in hydrochloric acid buffer solution (pH = 1.2) as a simulated gastric medium.

## Materials and Methods

### Materials

CS (medium molecular weight, degree of deacetylation­ = 85%) and sodium montmorillonite (Na-MMT, with the specific surface area of 20–40 m^2^/g and cation exchange capacity of 30 meq/100 g) were procured from Sigma-Aldrich (USA). Dialysis membrane bag (with a molecular cut-off of 12400) was also supplied from Sigma-Aldrich (USA). Acrylic acid (AA), acrylamide (AAm), PVP (the average molecular weight = 25000), N,N′-methylene bisacrylamide (MBA), ammonium persulfate (APS), and N,N,Nʹ,Nʹ-tetramethylethylenediamine (TEMED) were procured from Merck Company (Germany). Potassium chloride, hydrochloric acid, sodium acetate, acetic acid, sodium hydroxide, and acetone were also supplied from Merck Company (Germany). Ethanol (96%) was obtained from Mojallali Chemical Laboratories (Tehran, Iran). Other reagents and chemicals were of analytical grade and all solutions were prepared using distilled water.

#### Preparation of semi-IPN CS-g-poly(AA-co-AAm)/PVP/

##### MMT superabsorbent nanocomposite

First, CS (0.6 g) was completely solubilized in 20 mL acetic acid (1% v/v) aqueous solution in a 250 mL round bottom flask using a mechanical stirrer. Then, a suspension of MMT (3-18 wt%, with respect to CS) in 10 mL acetic acid (1% v/v) aqueous solution was prepared by sonication at 50 W power for 5 minutes. This suspension was then added into the flask containing sticky pasty like solution of CS and stirred continuously. Temperature of the reaction mixture was increased to 40°C by a water bath, and then 0.8 g of AAm and 0.045 g of PVP were poured into the flask, while stirring. After reaction mixture was purged with nitrogen gas for 30 minutes to remove dissolved oxygen, given amounts of partially neutralized AA (50%, 3.2 mL) and MBA (0.83 wt%, with respect to CS) were added into the reaction mixture. Thereafter, 5 mL of APS aqueous solution (6.6 wt%, with respect to CS) was poured into the reaction flask and after 5 minutes, 100 µL of TEMED aqueous solution (20% v/v) as catalyst was introduced. The reaction mixture was stirred throughout the polymerization process and its temperature was maintained at 40°C, while purging with nitrogen gas. After gelation process was completed, final product was cooled to room temperature. Then, it was cut into small pieces and submerged in excess ethanol for 24 hours to eliminate any unreacted monomers and oligomers. The extracted gel was freeze-dried under vacuum at -170°C. The dried gel particles was then milled and sifted through 40-80 mesh sieves for subsequent analyses. A neat semi-IPN CS-g-poly(AA-co-AAm)/PVP hydrogel sample was also synthesized similarly for comparison purposes.

### Preparation of acetate (pH = 4) and hydrochloric acid (pH = 1.2) buffer solutions

To prepare hydrochloric acid-potassium chloride buffer solution (pH = 1.2), aqueous solutions of hydrochloric acid and potassium chloride with the same volumes (250 mL) and concentrations (0.4 M) were first prepared. Then, hydrochloric acid solution was mixed gradually with 250 mL potassium chloride solution in a beaker using a magnetic stirrer. During this process, pH value of the aqueous solution was continuously being controlled by a pH meter and finally it was adjusted to pH = 1.2 by addition of diluted sodium hydroxide aqueous solution (0.1 M) if necessary. In the case of acetic acid-sodium acetate buffer solution (pH = 4), a similar procedure was also conducted except that aqueous solutions of acetic acid (0.169 M) and sodium acetate (0.029 M) were used as buffer solution constituents.

### Preparation of drug-loaded hydrogels

CAM was loaded in the hydrogel samples by a simple technique of swelling-loading. First, pre-determined amount of CAM (0.5 g) was slowly dissolved in 100 mL acetate buffer solution (pH = 4) using a magnetic stirrer. Then, freeze-dried sample of hydrogel (0.1 g) (40-80 mesh) was submerged entirely in the CAM aqueous solution. The prepared system was incubated at room temperature (25°C) and stirred continuously for 24 hours. Thereafter, the hydrogel sample was withdrawn from the solution and dried completely in a vacuum oven at 60°C. Afterwards, CAM-loaded hydrogel sample was freeze-dried under vacuum at -170°C. The supernatant liquid was collected to determine encapsulation efficiency (EE) and loading content (LC) of CAM (λ_max_ = 284.6 nm) using a UV-vis spectrometer. The calibration curve of CAM in acetate buffer solution was used to determine its concentration in the supernatant liquid, and then EE and LC were calculated using the following equations:

(1) $$EE=\frac{M_{t}-M_{f}}{M_{t}}$$

(2) $$LC=\frac{M_{t}-M_{f}}{M_{n}}$$

Where M_t_ corresponds to the total weight of CAM; M_f_ represents the free amount of CAM in the supernatant liquid; and M_n_ shows the weight of CAM-loaded hydrogel sample after freeze-drying.

### Characterizations

FTIR spectra of the materials were acquired using a FTIR spectrometer (Bruker, Tensor 27 spectrophotometer, Germany). The powdered dry materials were thoroughly mixed with KBr, pressed into a pellet, and then FTIR spectra of these pellets were recorded in the wavenumber range of 400-4000 cm^-1^. Thermogravimetric analysis (TGA) was conducted on the hydrogel samples by means of a thermal gravimetric analyzer (TGA/DSC-1, Mettler Toledo) under nitrogen atmosphere from 47°C to 610°C at a heating rate of 10°C/min. To determine the amount of drug released from the prepared formulations and also to designate λ_max_ of CAM in different buffer solutions including hydrochloric acid (pH = 1.2) and acetate (pH = 4), UV-vis spectroscopy was performed using a UV-vis spectrophotometer 1700, Shimadzu. Surface morphology of MMT, hydrogel samples and also drug delivery formulations was studied using a field emission scanning electron microscope (FE-SEM) system (MIRA3 FEG-SEM, Tescan, Czech).

### Evaluation of properties

#### Measurement of grafting efficiency and grafting ratio

Grafting efficiency (GE) defines the percentage of the grafted polymer in the stock polymer, while grafting ratio (GR) is assigned to the percentage of the grafted copolymer compared to the stock polymer. To determine GE and GR of the prepared hydrogel samples, certain amount of dry hydrogel sample (W_2_) was submerged in excess acetone for 24 hours to eliminate any unreacted monomers and oligomers. Then, it was withdrawn from the acetone, dried in a vacuum oven at 60°C for 24 hours, and weighed, accurately (W_3_). Finally, percentage of GE (%) and GR (%) was determined using the equation 3 and equation 4, respectively.^22,23^

(3) $$GE(\%)=\frac{w_{3}-w_{1}}{w_{2}-w_{1}}\times 100$$

(4) $$GR(\%)=\frac{w_{3}}{w_{1}}\times 100$$

W_1_ (g) is the weight of CS.

#### Swelling kinetics measurement

To investigate swelling kinetics of the hydrogels in distilled water, hydrochloric acid (pH = 1.2) and acetate (pH = 4) buffer solutions, gravimetric method was sued. First, certain amount of dry hydrogel sample (W_d_) (40-80 mesh) was added into a cylindrical steel screen (100 mesh), and then it was submerged in 100 mL distilled water or buffer solution at 25°C. At specified time intervals, this system was removed from the swelling medium and the net weight of swollen hydrogel sample (W_t_) was recorded after blotting the excess surface water with a filter paper. These measurements were continued till the weight of the swollen hydrogel sample remained at a constant value (W_eq_), indicating an equilibrium swelling state. All experiments were done in triplicate and eventually average values of the obtained data were recorded. Finally, equation 5 and equation 6 were used to calculate swelling capacity (Q_t_) and equilibrium swelling capacity (Q_eq_) of the hydrogel sample:

(5) $$Q_{t}(g/g)=\frac{w_{t}-w_{d}}{w_{d}}$$

(6) $$Q_{eq}(g/g)=\frac{w_{eq}-w_{d}}{w_{d}}$$

##### Rheological analysis

In order to evaluate viscoelastic properties of the hydrogels, mechanical responses of CS-g-poly(AA-co-AAm)/PVP and CS-g-poly(AA-co-AAm)/PVP/MMT samples versus oscillatory frequencies were recorded using a Anton Paar rheometer (MCR301, Germany) at 25°C equipped with parallel plates of 25 mm diameter and gap of 1 mm. The linear viscoelastic (LVE) region, demonstrating independent behavior of storage modulus (Gʹ) and loss modulus (Gʺ) versus applied strain amplitude, was first determined by strain sweep tests at frequency of ω = 10 Hz. The results showed that Gʹ and Gʺ values of hydrogel samples possess an independent behavior versus the applied strain in the deformation range below 0.5%, indicating LVE region. Thereafter, to assess viscoelastic behavior of the hydrogels, frequency sweep tests were executed at a constant strain (γ = 0.5) within angular frequency range of 0.1 to 100 Hz.

##### In vitro drug release studies

Briefly, 0.5 g of freeze-dried CAM-loaded hydrogel sample was poured into a dialysis membrane bag with both ends tied together. It was immersed in 100 mL hydrochloric acid buffer solution (pH = 1.2) as simulated gastric fluid. The system was incubated at 37°C with slight agitation during the experiment. At pre-determined time intervals, 5 mL of the release medium was withdrawn and replaced with equivalent volume of fresh buffer to keep the volume constant. The ultraviolet absorption of the released drug was measured in sampled solutions by UV-vis spectroscopy at λ_max_ = 283.8 nm. Then, the amount of drug in solution was determined using a calibration curve of CAM in hydrochloric acid buffer solution. Three replications were done for all release experiments, and finally average values were considered to calculate cumulative release of CAM. The accumulative release of drug was obtained using equation 7.^24^

(7) $$E=\frac{V_{E}\sum_{1}^{n-1}C_{i}+V_{0}C_{n}}{m_{0}}\times 100$$

Where *E* signifies the accumulative release (%) of CAM and V_E_ and V_0_ (mL) relate to the sampling volume and the initial volume of the release media, respectively. C_i_ and C_n_ attribute to the drug concentrations (mg/mL), i and n show the sampling times, and m_0_ ascertains the mass of the drug in the hydrogel samples (mg).

## Results and Discussion

### Synthesis mechanism of semi-IPN superabsorbent nanocomposite

**Scheme 1:**
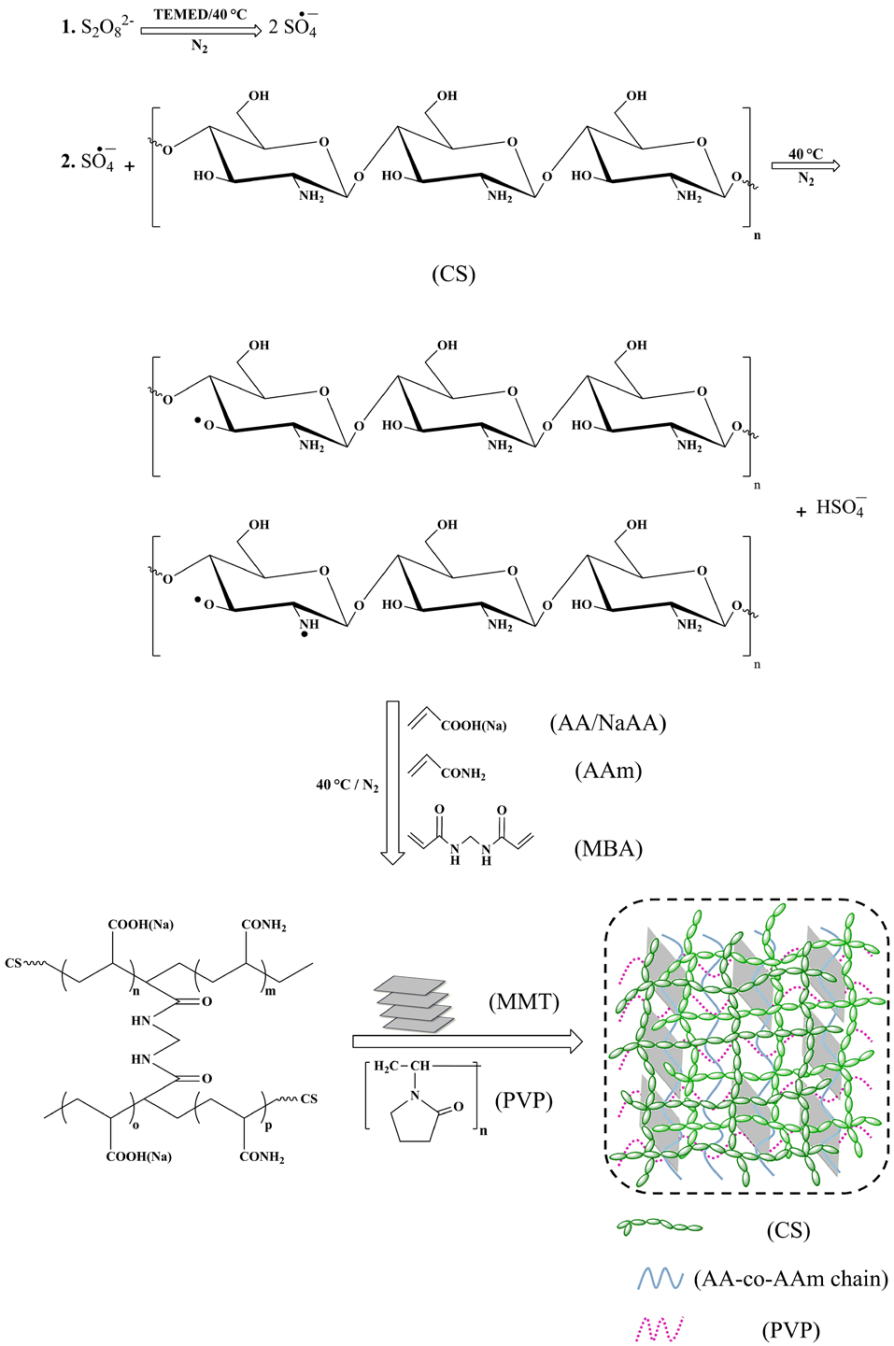
Proposed graft copolymerization mechanism for synthesis of semi-IPN CS-g-poly (AA-co-AAm)/PVP/MMT superabsorbent nanocomposite.

Graft copolymerization reaction and PVP interpenetration through hydrogel network were carried out at the same time in an aqueous solution containing an initiator (APS), crosslinking agent (MBA), and filler (MMT) (Scheme 1). Initially, APS molecules in the presence of TEMED as catalyst dissociate to produce sulfate anion-radicals. The active radicals produced by this way, generate CS macro radicals by abstraction of hydrogen atoms from its hydroxyl or amine functional groups. Then, AA and AAm monomers, which are closer to the CS macro radicals, can accept the active radical center, resulting in the growing graft copolymer chains (propagation step). As the propagation of graft copolymer chains proceeds, the vinyl groups of MBA may couple synchronously with active radical centers on the CS backbone, copolymer chains, and graft copolymer chains to build a crosslinked structure. At the same time, non-covalent hydrogen bonding interactions between PVP as an interpenetrating polymer and functional groups of crosslinked hydrogel network form a semi-IPN hydrogel network. Final network structure of the semi-IPN superabsorbent nanocomposite is made in the presence of MMT layers, which act as physical crosslinking agent.^25^

The amounts of GE (%) and GR (%) for CS-g-poly(AA-co-AAm)/PVP and CS-g-poly(AA-co-AAm)/PVP/MMT samples were found to be 96.5, 2271, and 99.15 and 2296, respectively. CS-g-poly(AA-co-AAm)/PVP/MMT possessed higher amounts of GE (%) and GR (%) compared with CS-g-poly(AA-co-AAm)/PVP. This is due to the hydrogen-bonding interactions between hydroxyl groups of introduced MMT and functional groups of the acrylate-based monomers, which make an improvement in the final hydrogel network structure.

### FTIR spectra analysis

**Figure 1:**
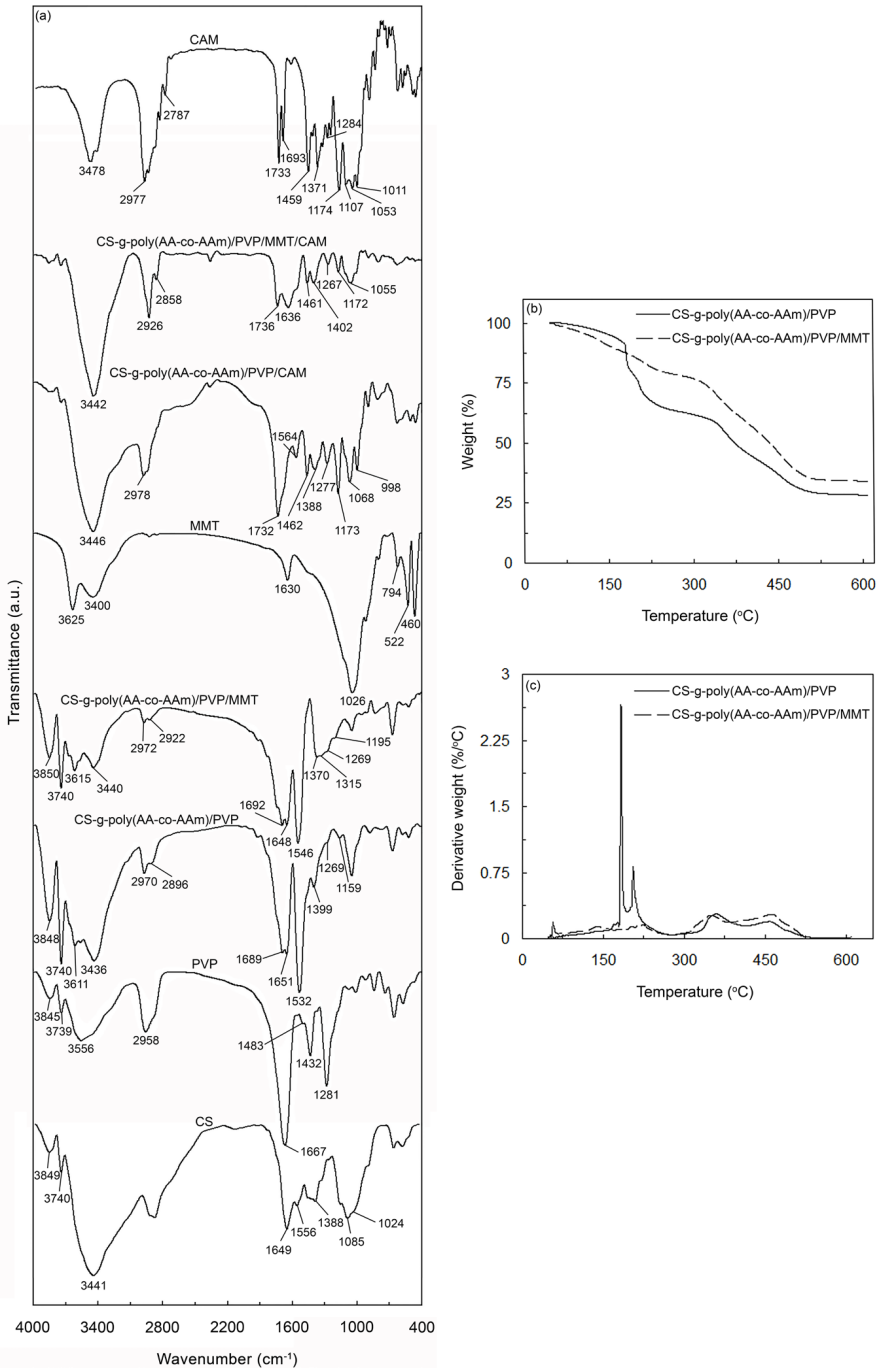
FTIR spectra of CS, MMT, PVP, CAM, CS-g-poly(AA-co-AAm)/PVP, CS-g-poly(AA-co-AAm)/PVP/MMT, CS-g-poly(AA-co-AAm)/PVP/CAM, and CS-g-poly(AA-co-AAm)/PVP/MMT/CAM (a), and TGA (b) and DTG (C) curves of CS-g-poly(AA-co-AAm)/PVP and CS-g-poly(AA-co-AAm)/PVP/MMT (with 13 wt% MMT).

The FTIR spectra of CS, MMT, PVP, CAM, CS-g-poly(AA-co-AAm)/PVP, CS-g-poly(AA-co-AAm)/PVP/MMT, CS-g-poly(AA-co-AAm)/PVP/CAM, and CS-g-poly(AA-co-AAm)/PVP/MMT/CAM have been depicted in Figure 1a. As shown in Figure 1a for CS, the relatively broad band emerged at 3441 cm^-1^ is related to the stretching vibration modes of O–H and N–H groups as well as intermolecular hydrogen-bonding interactions of polysaccharide moieties.^24^ Also, the stretching vibration modes of carbonyl group (C=O) of amide I, N–H bond, –NHCO of amide III, C_3_–OH, and C_6_–OH of CS were appeared at 1649 cm^-1^, 1556 cm^-1^, 1388 cm^-1^, 1085 cm^-1^, and 1024 cm^-1^, respectively.^26^ According to the FTIR spectrum of MMT (Figure 1a), the characteristic absorption bands related to the stretching vibrations of Si–O–Al and Si–O–Si groups were appeared at 794 cm^-1^ and 1026 cm^-1^, respectively, while their bending vibration modes were observed at 522 cm^-1^ and 460 cm^-1^, respectively. Also, the absorption band at 1630 cm^-1^ is due to the bending mode of hydroxyl group of the adsorbed water. Moreover, the broad peaks at 3400 cm^-1^ and 3625 cm^-1^ are assigned to the stretching modes of –OH groups of water and –OH groups in the inner structure of MMT.^24,27^ According to the FTIR spectrum of PVP (Figure 1a), the sharp peak at 1667 cm^-1^ is attributed to the stretching vibration of C=O. Also, the characteristic absorption bands centered at 1281 cm^-1^, 1432 cm^-1^, and 1483 cm^-1^ wavenumbers are corresponded to the C–N stretching vibration mode. Moreover, the absorption band appeared at 2958 cm^-1^ is related to the stretching vibration of CH_2_ groups.^28-30^ FTIR spectrum of pure CAM (Figure 1a) shows characteristic peaks at 1693 cm^-1^ and 1733 cm^-1^, which are corresponded to the stretching vibration of ketone and lactone carbonyl groups, respectively. An absorption band emerged at 1459 cm^-1^ is due to the stretching vibration of N–C bonds of amine groups. The peaks observed from 2780 cm^-1^ to 3000 cm^-1^ wavenumbers are also assigned to the stretching vibration modes of C–C bonds of alkane groups. Moreover, an absorption band emerged at 3478 cm^-1^ as a relatively sharp peak is attributed to the intermolecular hydrogen bonds between hydroxyl groups. Additionally, the stretching vibration modes of –C–O and CH_2_ groups were appeared in the wavenumber ranges of 1000-1200 cm^-1^ and 1340-1400 cm^-1^, respectively.^31^ According to the FTIR spectra of CS-g-poly(AA-co-AAm)/PVP, CS-g-poly(AA-co-AAm)/PVP/MMT, CS-g-poly(AA-co-AAm)/PVP/CAM, and CS-g-poly(AA-co-AAm)/PVP/MMT/CAM (Figure 1a), the stretching vibration mode of carboxamide and bending vibration of N–H in amide group have overlapped together, which were emerged at 1689 cm^-1^, 1692 cm^-1^, 1732 cm^-1^, and 1736 cm^-1^, respectively. Also, in the case of CS-g-poly(AA-co-AAm)/PVP/CAM and CS-g-poly(AA-co-AAm)/PVP/MMT/CAM (Figure 1a), the stretching vibration modes of carboxamide groups observed at respectively 1732 cm^-1^ and 1736 cm^-1^, have overlapped with stretching vibration modes of ketone and lactone carbonyl groups of CAM. The overlapped asymmetric stretching vibrations of the carboxylate groups of CS-g-poly(AA-co-AAm)/PVP, CS-g-poly(AA-co-AAm)/PVP/MMT, CS-g-poly(AA-co-AAm)/PVP/CAM, and CS-g-poly(AA-co-AAm)/PVP/MMT/CAM and vibration mode of carbonyl group of amide I of CS were appeared at 1651 cm^-1^, 1648 cm^-1^, 1564 cm^-1^, and 1636 cm^-1^, respectively. Also, the dual absorption bands in FTIR spectra of CS-g-poly(AA-co-AAm)/PVP, CS-g-poly(AA-co-AAm)/PVP/MMT, CS-g-poly(AA-co-AAm)/PVP/CAM, and CS-g-poly(AA-co-AAm)/PVP/MMT/CAM emerged at 1532 cm^-1^, 1399 cm^-1^, and 1546 cm^-1^, 1370 cm^-1^, and 1462 cm^-1^, 1388 cm^-1^, and 1461 cm^-1^ and 1402 cm^-1^, respectively, are attributed to the symmetric stretching vibration modes of carboxylate groups. The peaks in the region of 1150–1350 cm^-1^ are related to the stretching modes of C–N and C–O groups as well as bending mode of O–H bond. Moreover, two peaks appeared within the range of 2850–2980 cm^-1^ in FTIR spectra of CS-g-poly(AA-co-AAm)/PVP, CS-g-poly(AA-co-AAm)/PVP/MMT, CS-g-poly(AA-co-AAm)/PVP/CAM, and CS-g-poly(AA-co-AAm)/PVP/MMT/CAM are due to the combined stretching vibration modes of CH_2_ groups in both AA and AAm moieties. Furthermore, the characteristic absorption peaks of O–H and N–H groups were emerged as overlapped broad and intense bands between 3400 cm^-1^ and 3600 cm^-1^. ^26,32^ From FTIR spectra of CS-g-poly(AA-co-AAm)/PVP, CS-g-poly(AA-co-AAm)/PVP/MMT, CS-g-poly(AA-co-AAm)/PVP/CAM, and CS-g-poly(AA-co-AAm)/PVP/MMT/CAM it is evident that the peaks related to the stretching vibrations of N–H (1556 cm^-1^ and 1388 cm^-1^) and C_3_–OH (1085 cm^-1^) groups of CS, have been disappeared after reaction. These findings revealed that –NH_2_, –NHCO, and –OH groups of CS have been effectively participated in grafting reaction with acrylate-based monomers.^33^ Moreover, the stretching vibration mode of carbonyl group of PVP (1667 cm^-1^) has emerged at the higher wavenumbers of 1689 cm^-1^, 1692 cm^-1^, 1732 cm^-1^, and 1736 cm^-1^ in respectively FTIR spectra of CS-g-poly(AA-co-AAm)/PVP, CS-g-poly(AA-co-AAm)/PVP/MMT, CS-g-poly(AA-co-AAm)/PVP/CAM, and CS-g-poly(AA-co-AAm)/PVP/MMT/CAM, and has overlapped with the corresponding peaks of carboxamide and N–H groups. This phenomenon results from the strong H-bonding interactions, which are formed between carboxamide and carbonyl groups. In addition, the characteristic absorption bands of C–N groups of PVP have been appeared with slight shift in FTIR spectra of CS-g-poly(AA-co-AAm)/PVP, CS-g-poly(AA-co-AAm)/PVP/MMT, CS-g-poly(AA-co-AAm)/PVP/CAM, and CS-g-poly(AA-co-AAm)/PVP/MMT/CAM. These results imply that PVP chains have successfully interpenetrated through hydrogel network structure by hydrogen-bonding interactions.^28^ According to FTIR spectra of CS-g-poly(AA-co-AAm)/PVP/MMT and CS-g-poly(AA-co-AAm)/PVP/MMT/CAM, it can be seen that characteristic absorption band of MMT (1026 cm^-1^) has appeared with slight shift in wavenumbers, indicating that MMT has successfully been incorporated into hydrogel network. Moreover, the characteristic peaks of CAM have emerged with slight shift in FTIR spectra of CS-g-poly(AA-co-AAm)/PVP/CAM and CS-g-poly(AA-co-AAm)/PVP/MMT/CAM. This implies that CAM has been successfully loaded in the hydrogel network. These findings provide a strong evidence for successful synthesis of CS-g-poly(AA-co-AAm)/PVP, CS-g-poly(AA-co-AAm)/PVP/MMT and also loading of CAM in the hydrogel network.

### Thermogravimetric analysis

Figure 1b and 1c show TGA and differential TGA (DTG) curves of CS-g-poly(AA-co-AAm)/PVP and CS-g-poly(AA-co-AAm)/PVP/MMT (with 13 wt% MMT), respectively. As shown in Figure 1b and 1c, CS-g-poly(AA-co-AAm)/PVP exhibited four distinct decomposition stages from 47°C to 610°C, while decomposition process of CS-g-poly(AA-co-AAm)/PVP/MMT superabsorbent nanocomposite accomplished within five stages. As the temperature was increased to 200°C, a severe weight loss of 23.07% was occurred for CS-g-poly(AA-co-AAm)/PVP, which is corresponded to the loss of moisture present in the sample. A minor weight loss (3.52%) was observed for CS-g-poly(AA-co-AAm)/PVP/MMT when the temperature was increased from 47°C to 100°C, which was due to the dehydration of adsorbed water, interlayer water, and coordinated water to exchangeable cations of MMT.^34^ With increasing temperature to 180°C, the moisture present in CS-g-poly(AA-co-AAm)/PVP/MMT evaporated gradually, which in turn induced a weight loss of 8.97%. The weight losses within the temperature ranges of 200–328°C and 180–337°C for respectively CS-g-poly(AA-co-AAm)/PVP (22.02%) and CS-g-poly(AA-co-AAm)/PVP/MMT (18.56%) are ascribed to the complex processes including dehydration of saccharide rings and breaking of glycosidic bonds (C–O–C) in CS chain.^33^ The major weight losses of 29.74% and 31.41% were also found for CS-g-poly(AA-co-AAm)/PVP and CS-g-poly(AA-co-AAm)/PVP/MMT samples, respectively, which were occurred respectively within the temperature ranges of 328–420°C and 337–440°C. Dissociation of copolymer chains and thermal decomposition of their carboxyl and amide groups are responsible for these weight losses, which are usually accompanied by emission of ammonia and CO_2_ gases.^35^ The last decomposition stages for CS-g-poly(AA-co-AAm)/PVP and CS-g-poly(AA-co-AAm)/PVP/MMT started at 420°C and 440°C, respectively, and extended to about 610°C. The weight losses of CS-g-poly(AA-co-AAm)/PVP and CS-g-poly(AA-co-AAm)/PVP/MMT within the corresponding temperature ranges were obtained as 32.87% and 30.33%, respectively. These weight losses result from several processes, which can be elucidated as follows. The removal of water molecules, which are formed during association of two neighboring carboxylic acid groups on the polymer chain, is the first factor. Thermal decomposition of copolymer chain and also destruction of the final crosslinked network structure of hydrogel are other important factors, which induce a significant reduction in total weight of hydrogel sample.^34^ From these results, it can be concluded that incorporation of MMT into hydrogel network causes a lower thermal decomposition rate and also less total weight loss over the whole temperature range from 47°C to 610°C. The heat barrier effect of MMT layers is the main reason for this phenomenon, which hinders diffusion of oxygen and volatile thermo-oxidation products throughout the hydrogel composite network.^36^ Besides, the additional physical crosslinkages within hydrogel network made by introduced MMT build a firm three-dimensional hydrogel structure with good thermal stability.

### Surface morphology studies

**Figure 2:**
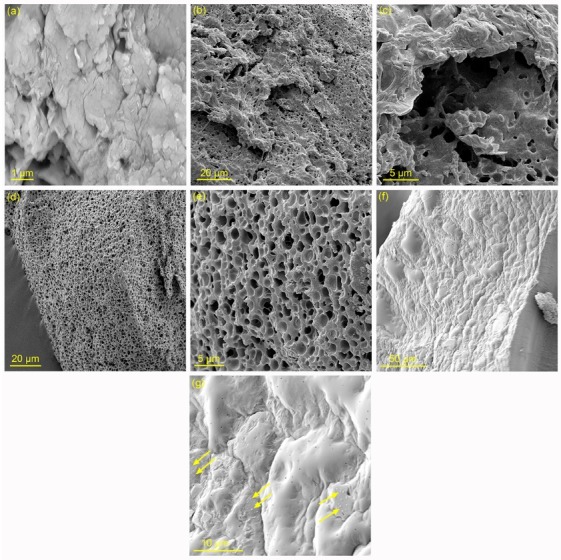
SEM images of MMT (a), CS-g-poly(AA-co-AAm)/PVP (b) and (c), CS-g-poly(AA-co-AAm)/PVP/MMT (with 13 wt% MMT) (d) and (e), and freeze-dried CS-g-poly(AA-co-AAm)/PVP/MMT/CAM (f) and (g).

To study surface morphology changes in the presence of MMT, SEM analysis was performed on the hydrogel samples. Scanning electron micrographs of the prepared materials have been depicted in Figure 2a-g. In order to prevent degradation of CAM and also to provide a clear surface morphology, freeze-drying process was used on the hydrogel samples before SEM analysis. As shown in SEM image of MMT (Figure 2a), the aggregated MMT layers have formed more dense layered structure. SEM images of CS-g-poly(AA-co-AAm)/PVP (Figure 2b and 2c) show a coarse porous surface morphology. These structures result from the additional H-bonding interactions between PVP chains and functional groups of graft copolymer chains, increase contact surface area and so can improve swelling rate and water absorption capacity. In comparison with CS-g-poly(AA-co-AAm)/PVP sample, superabsorbent nanocomposite shows a highly porous structure with interlinked channels (Figure 2d and 2e). These interlinked porous structures made by physical crosslinkages in the presence of MMT provide high amount of available pores to be occupied by water molecules. Therefore, CS-g-poly(AA-co-AAm)/PVP/MMT sample will have greater water absorption capacity compared with neat hydrogel. SEM images of CAM-loaded superabsorbent nanocomposite (Figure 2f and 2g) show more dense surface morphology with low porosity. As seen in Figure 2g, some of the CAM crystals migrated to the surface of hydrogel has emerged as white dots (as shown by arrow signs). In spite of the fact that lower porosity may reduce drug release rate, but CAM crystals on the hydrogel surface can induce a burst release of drug during initial time periods after drug delivery formulation was immersed in the swelling medium.

### Swelling kinetic studies

**Figure 3:**
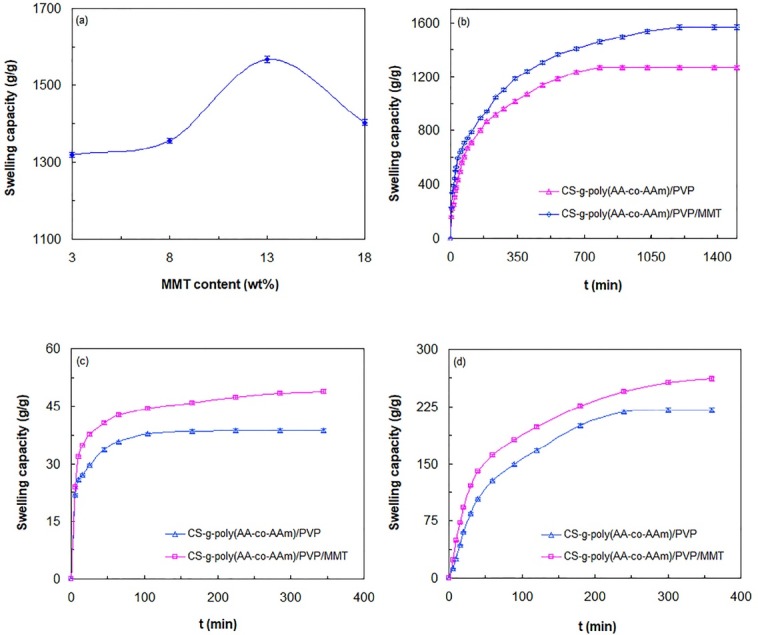
The equilibrium swelling capacity of CS-g-poly(AA-co-AAm)/PVP/MMT samples at different MMT contents (a), swelling kinetic diagrams of CS-g-poly(AA-co-AAm)/PVP and CS-g-poly(AA-co-AAm)/PVP/MMT (with 13 wt% MMT) samples in distilled water (b), hydrochloric acid (pH=1.2) (c), and acetate (pH=4) buffer solutions (d).

To evaluate the effect of MMT content on the water absorption capacity of hydrogel sample, equilibrium water absorption capacity of CS-g-poly(AA-co-AAm)/PVP/MMT hydrogels containing various MMT contents was measured in distilled water (Figure 3a). As shown in Figure 3a, with increasing MMT content from 3 wt% to 13 wt% swelling capacity increases substantially so that maximum swelling capacity of 1568 g/g was obtained at 13 wt% MMT content. When MMT content rises beyond 13 wt%, a considerable reduction in the swelling capacity is occurred and equilibrium water absorption capacity reaches 1403 g/g at 18 wt% MMT content. These results can be interpreted by the following facts. When MMT content is lower than 13 wt%, electrostatic repulsive forces between hydroxyl groups of the incorporated MMT and carboxylate anions of the polymeric matrix are dominant phenomenon, which make a great expansion in hydrogel network. At this condition, the enlarged available voids within hydrogel network can be occupied by higher amount of water molecules, resulting in the increased swelling capacity. Moreover, due to the hydrophilic character of hydroxyl groups of MMT, osmotic pressure difference between swelling medium and hydrogel network increases dramatically, leading to the enhanced swelling capacity. Further increase in MMT content up to 18 wt% brings a severe reinforcement in physical crosslinking density and so a reduction in water absorption capacity. In addition, hydrophilicity of the polymeric matrix of hydrogel is higher than that of MMT, which makes it most responsible for water absorption of the hydrogel. Hence, at higher MMT contents (greater than 13 wt%), the ratio of polymeric matrix in the hydrogel composition decreases and thus swelling capacity decreases.^32^ Figure 3b shows swelling kinetic curves of CS-g-poly(AA-co-AAm)/PVP and CS-g-poly(AA-co-AAm)/PVP/MMT (with 13 wt% MMT) samples in distilled water. According to Figure 3b, a similar swelling kinetic trend is observed for both hydrogel samples in which water absorption capacity increases rapidly initially, and then its growth rate decreases slowly and remains almost constant till it reaches to an equilibrium state. According to Figure 3b, the equilibrium swelling capacity of CS-g-poly(AA-co-AAm)/PVP (1270 g/g) and CS-g-poly(AA-co-AAm)/PVP/MMT (1568 g/g) was acquired after 780 minutes and 1200 minutes, respectively. These results indicated that introduced MMT makes a substantial improvement in water absorption capacity. Moreover, in the presence of MMT, penetration of water molecules into hydrogel, due to the interlinked porous structures within hydrogel network, occurs with a slow rate, causing a delay in the equilibrium swelling time. Figure 3c and 3d exhibit swelling kinetic curves of CS-g-poly(AA-co-AAm)/PVP and CS-g-poly(AA-co-AAm)/PVP/MMT samples in hydrochloric acid (pH = 1.2) and acetate (pH = 4) buffer solutions, respectively. As shown in Figure 3c and 3d, both hydrogel samples in different buffer solutions follow a similar swelling kinetic trend, which was the same as that in distilled water. According to Figure 3c, in hydrochloric acid buffer solution, CS-g-poly(AA-co-AAm)/PVP and CS-g-poly(AA-co-AAm)/PVP/MMT samples reached to own equilibrium swelling capacity values of respectively 38.7 g/g and 48.9 g/g after 225 min and 345 minutes, respectively. In acetate buffer solution (Figure 3d), the equilibrium water absorption capacity values of CS-g-poly(AA-co-AAm)/PVP (221 g/g) and CS-g-poly(AA-co-AAm)/PVP/MMT (261.5 g/g) samples were achieved within 300 minutes and 360 minutes, respectively. These findings possessed good compliance with the results obtained previously in distilled water. According to the obtained results, it is evident that hydrogel samples in acetate buffer solution possess higher swelling capacity compared with the hydrochloric acid buffer solution. This can be explained as follows. Amine functional groups on the CS backbone are weak base (pK_a_ = 6.5) and carboxylic acid groups on poly AA chains have relatively strong acidic nature with pK_a_ of about 4.7. In acidic solution of acetate buffer (pH = 4), the high electrostatic repulsions among protonated ammonium groups (–NH_3_^+^) cause an expansion in hydrogel network and thus enable hydrogel to swell more. However, in highly acidic solution of hydrochloric acid buffer (pH = 1.2), the excess amount of Cl- counterions shield the ammonium charges and prevent effective cation-cation repulsions. This condition makes a shrinkage in hydrogel network, and thus swelling capacity decreases.^37,38^

The swelling kinetics of the hydrogel samples were studied by fitting pseudo-second order equation (equation 8) to the experimental swelling data.

(8) t/W=A+Bt

(9) $$A=\frac{1}{k_{s}W_{\infty }^{2}}$$

(10) $$B=1/W_{\infty }$$

**Figure 4:**
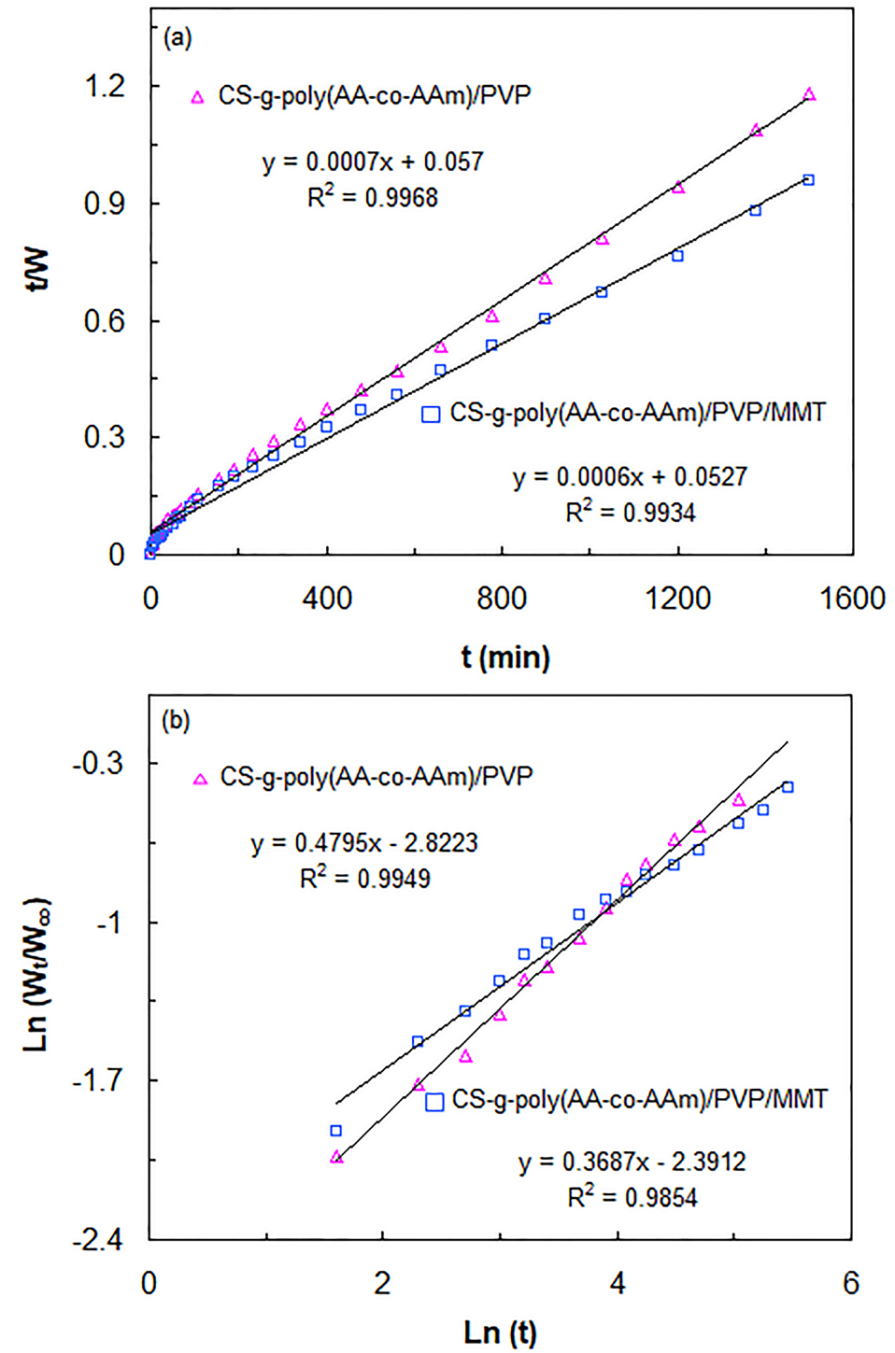
Plots of t⁄W versus t (a) and plots of Ln (W_t_⁄W_∞_) versus Ln (t) for CS-g-poly(AA-co-AAm)/PVP and CS-g-poly(AA-co-AAm)/PVP/MMT samples (b).

Where W (g/g) corresponds to the swelling capacity at a moment time of t (min); the A factor is the primary swelling rate of the hydrogel; k_s_ (g/g.min) attributes to the swelling rate constant; and W_∞_ (g/g) designates theoretical equilibrium water absorption capacity.^39^ The plots of t/w versus *t* for CS-g-poly(AA-co-AAm)/PVP and CS-g-poly(AA-co-AAm)/PVP/MMT samples have been depicted in Figure 4a. The amounts of W_∞_and k_s_ were calculated from the slope and intercept of the plotted straight lines, respectively, and the obtained data were provided in Table 1. The obtained results showed that the theoretical equilibrium swelling capacity values of CS-g-poly(AA-co-AAm)/PVP (1428.57 g/g) and CS-g-poly(AA-co-AAm)/PVP/MMT (1666.66 g/g) samples are very close to their corresponding experimental values. Also, CS-g-poly(AA-co-AAm)/PVP possessed higher swelling rate constant compared with CS-g-poly(AA-co-AAm)/PVP/MMT, demonstrating that swelling process of neat hydrogel sample is occurred more rapidly than that of superabsorbent nanocomposite. This phenomenon may be related to the tortuous porous pathways with interlinked channels within CS-g-poly(AA-co-AAm)/PVP/MMT network, which retard water diffusion process, extend the time needed to attain an equilibrium water absorption capacity, and therefore diminish swelling rate.

**Table 1:** The swelling and diffusion parameters for CS-g-poly(AA-co-AAm)/PVP and CS-g-poly(AA-co-AAm)/PVP/MMT samples

**Sample**	**K**_s_ **(g.min** ^-1^ **.g** ^-1^ **)**	W_∞_**(g.g**^-1^**)**	**n**	**k**
CS-g-poly(AA-co-AAm)/PVP	8.59 × 10^-6^	1428.57	0.4795	0.059
CS-g-poly(AA-co-AAm)/PVP/MMT	6.82 × 10^-6^	1666.66	0.3687	0.091

In order to study water diffusion mechanism of the hydrogel samples, the initial 60% of the fractional swelling data of the hydrogels was assessed using the following equation (equation 11).

(11) $${\text{W}}_{t}/W_{\infty }=kt^{n}$$

The equilibrium water absorption capacity is defined by W_∞_ (g/g) and swelling capacity at time t (min) is determined by W_t_ (g/g). The k parameter is proportionality constant and ascertains the type of water diffusion mechanism. When n = 0.5, water diffusion process is occurred by Fickian mechanism. For 0.5 < n < 1.0, water diffusion mechanism conforms to non-Fickian or anomalous transport behavior; for n = 1.0 water transport behavior follows case–II diffusion; and for n > 1, diffusion of water is controlled by supercase–II mechanism.^40^ The values of k and n for CS-g-poly(AA-co-AAm)/PVP and CS-g-poly(AA-co-AAm)/PVP/MMT samples are determined from the respectively slope and intercept of the straight lines obtained by Plotting Ln (W_t_/W_∞_) versus Ln (t) (Figure 4b). The amount of these parameters has been listed in Table 1. As depicted in Table 1, n values for CS-g-poly(AA-co-AAm)/PVP and CS-g-poly(AA-co-AAm)/PVP/MMT samples were found to be 0.4795 and 0.3687, respectively. These results indicated that water diffusion process in both hydrogel samples occurs by Fickian diffusion mechanism.

### Rheological measurements

**Figure 5:**
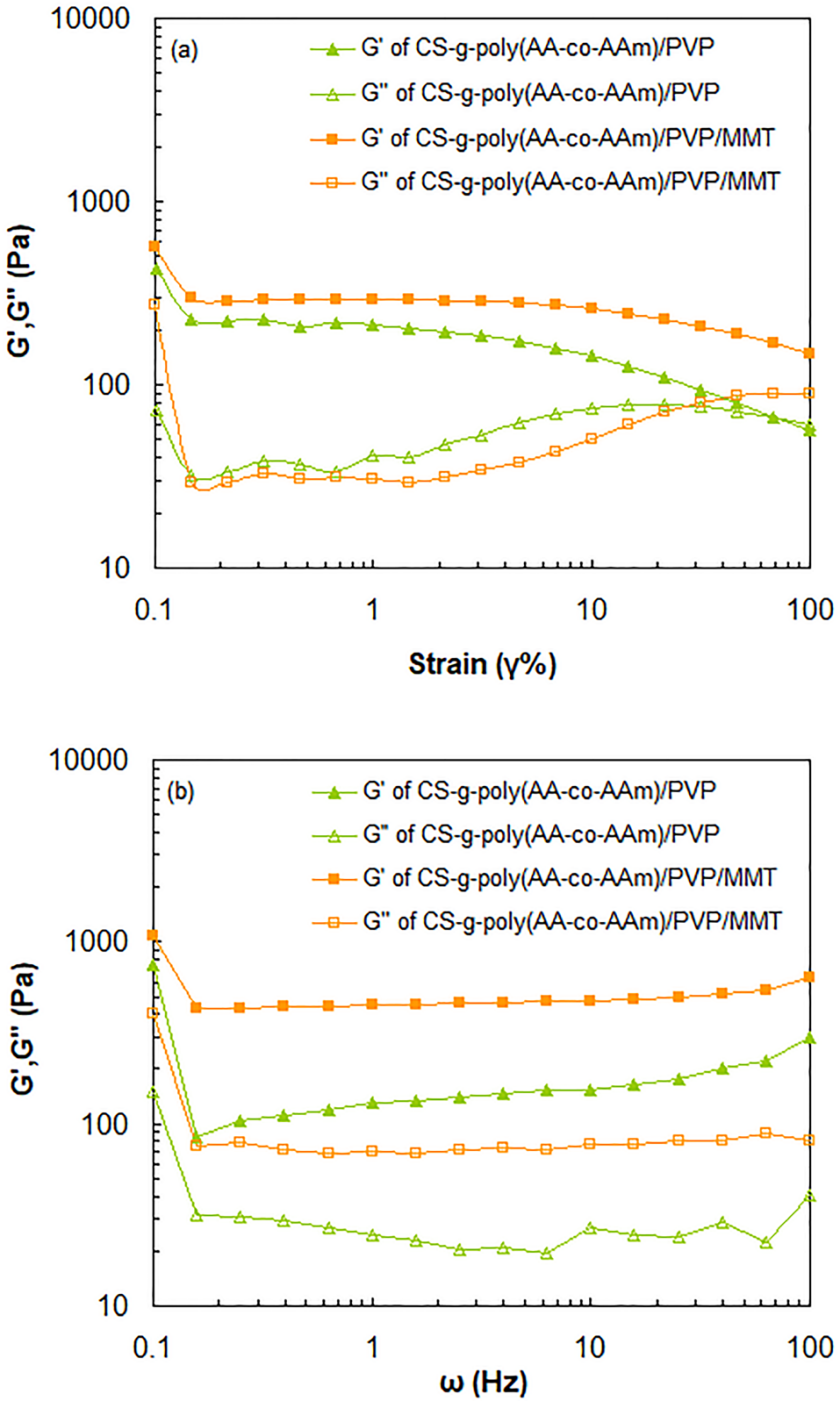
Strain dependence of the storage modulus (Gʹ) and loss modulus (Gʺ) at a constant angular frequency (ω = 10 Hz) (a) and frequency dependence of storage modulus (Gʹ) and loss modulus (Gʺ) at a constant strain (0.5%) for CS-g-poly(AA-co-AAm)/PVP and CS-g-poly(AA-co-AAm)/PVP/MMT samples (b).

One of the most important features of the hydrogels used in the field of controlled drug delivery formulations is the gel strength.^41^ The dynamic mechanical responses of the CS-g-poly(AA-co-AAm)/PVP and CS-g-poly(AA-co-AAm)/PVP/MMT samples were recorded as storage modulus (Gʹ) and loss modulus (Gʺ) within the angular frequency range of 0.1-100 Hz (Figure 5b). The linear viscoelastic (LVE) region in which the amount of applied strain does not affect Gʹ and Gʺ values, was first determined through execution of strain sweep tests on the hydrogels at constant frequency of ω = 10 Hz (Figure 5a). According to Figure 5a, in deformation ranges lower than 0.5%, Gʹ and Gʺ exhibit strain-independent behavior, demonstrating LVE region. Hence, deformation of 0.5% was ascertained as strain amplitude. Figure 5b shows the frequency dependence behavior of Gʹ and Gʺ values of hydrogel samples within the oscillatory frequency range of 0.1-100 Hz. However, in the frequency range of 1-10 Hz, Gʹ values of both hydrogel samples remained almost constant. This phenomenon demonstrates the frequency-independent feature of storage modulus of the hydrogels. As shown in Figure 5b for both hydrogel samples, the values of Gʹ were much higher than that of Gʺ values over the entire frequency range. This implies that hydrogel samples have a dominant elastic behavior compared to viscous one, which is a special character of the stable three-dimensional crosslinked hydrogel networks.^42^ Moreover, CS-g-poly(AA-co-AAm)/PVP/MMT superabsorbent nanocomposite possessed greater Gʹ values compared with CS-g-poly(AA-co-AAm)/PVP throughout the whole frequency range, indicating stiff hydrogel network of superabsorbent nanocomposite. Besides, Gʺ values of CS-g-poly(AA-co-AAm)/PVP/MMT from 0.1 to 100 Hz exhibited the loss of 79.97%, while in the case of CS-g-poly(AA-co-AAm)/PVP, the loss of Gʺ values within the corresponding frequency range was 72.98%. This is another evidence for stiff and strong gel framework of superabsorbent nanocomposite compared with the neat hydrogel. The strong network structure of superabsorbent nanocomposite results from the physical crosslinking effect of MMT, which forms a firm framework by keeping polymer chains together tightly and so improves mechanical strength of the hydrogel.

### In vitro drug release studies

The EE and LC of CAM for CS-g-poly(AA-co-AAm)/PVP/CAM and CS-g-poly(AA-co-AAm)/PVP/MMT/CAM drug delivery formulations were determined by the method mentioned previously in section 2.4. For CS-g-poly(AA-co-AAm)/PVP/CAM and CS-g-poly(AA-co-AAm)/PVP/MMT/CAM formulations the amounts of EE and LC were calculated as 25.63%, 79.81%, and 31.29% and 86.5%, respectively. According to the results, CS-g-poly(AA-co-AAm)/PVP/MMT/CAM formulation possessed higher amount of EE compared with CS-g-poly(AA-co-AAm)/PVP/CAM formulation. This is due to the hydrophilic character of the introduced MMT, which enhances swelling capacity of superabsorbent nanocomposite and so enables it to imbibe and preserve higher amount of CAM within own network. Moreover, H-bonding interactions between CAM molecules and hydroxyl groups of MMT create an attraction force, which promotes absorption of drug molecules from swelling medium and thus increases the amount of EE.

**Figure 6:**
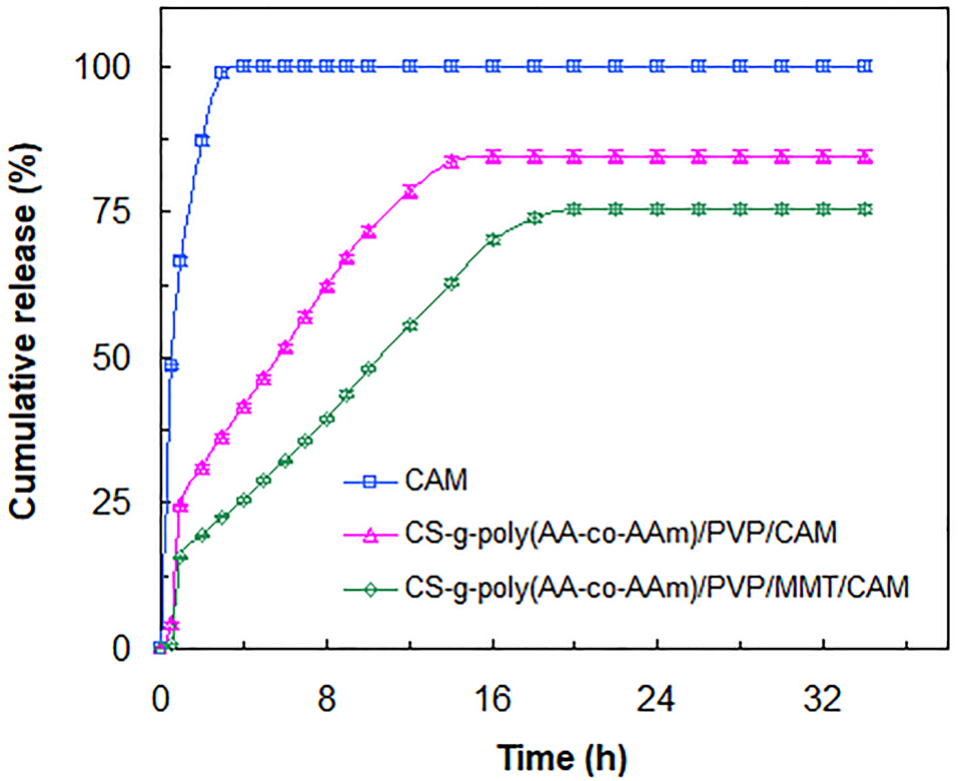
In vitro drug release patterns of pure CAM, CS-g-poly(AA-co-AAm)/PVP/CAM, and CS-g-poly(AA-co-AAm)/PVP/MMT/CAM formulations.

Figure 6 shows the in vitro drug release patterns of pure CAM, CS-g-poly(AA-co-AAm)/PVP/CAM, and CS-g-poly(AA-co-AAm)/PVP/MMT/CAM formulations in hydrochloric acid buffer (pH = 1.2) solution. According to Figure 6, pure CAM presented a quick rise in drug release rate compared with CS-g-poly(AA-co-AAm)/PVP/CAM and CS-g-poly(AA-co-AAm)/PVP/MMT/CAM formulations so that more than 98% drug released within 3 hours. This is due to the ease of solubility of CAM in hydrochloric acid buffer solution, which makes a rapid growth in drug release rate after pure CAM being immersed in release medium. In the case of CS-g-poly(AA-co-AAm)/PVP/CAM and CS-g-poly(AA-co-AAm)/PVP/MMT/CAM formulations (Figure 6), swollen hydrogel network acts as a barrier, which reduces drug release rate and so induces a sustained-release character. However, drug release patterns of CS-g-poly(AA-co-AAm)/PVP/CAM and CS-g-poly(AA-co-AAm)/PVP/MMT/CAM formulations showed burst release of drug in the first 3 hours of drug release period. This rapid release of drug at initial time periods is mainly attributed to the quick dissolution of the drug molecules existed in nearby to or on the surface of the CAM-loaded formulations. In spite of the fact that high concentration of drug at initial burst release may be helpful in prevention of bacterial resistance against therapeutic antibiotic, the burst release, due to the reduction of effective lifetime of drug, from the therapeutic point of view, is mostly undesirable.^14,24^ At the second phase of drug release patterns of CS-g-poly(AA-co-AAm)/PVP/CAM and CS-g-poly(AA-co-AAm)/PVP/MMT/CAM formulations, dissolution of the drug molecules in the inner porous structure of the hydrogel network made a slower release trend. During this step, as the hydrogel samples swell further, due to the continuous release of drug, concentration gradient between hydrogel network and drug release medium decreases. At this condition, drug release rate decreases gradually and remains almost constant once equilibrium swelling capacity is achieved. By comparing drug release patterns of CAM-loaded formulations, it can be found that CS-g-poly(AA-co-AAm)/PVP/MMT/CAM formulation liberates drug more slower than that of CS-g-poly(AA-co-AAm)/PVP/CAM formulation. Also, the time to achieve 50% release of drug (T_50%_) in CS-g-poly(AA-co-AAm)/PVP/CAM formulation was about 6 h, while in the case of CS-g-poly(AA-co-AAm)/PVP/MMT/CAM formulation, it took 10 hours to release 50% of drug. These findings revealed that CS-g-poly(AA-co-AAm)/PVP/MMT/CAM formulation possesses an effective sustain release property during the whole period of drug release. The main reason for this behavior is the presence of interlinked porous channels within superabsorbent nanocomposite network, which are formed by physical crosslinkages in the presence of MMT. The interlinked porous channels with more tortuous pathways in the CS-g-poly(AA-co-AAm)/PVP/MMT/CAM network hinder dissolution and diffusion of the CAM molecules, and so reduce drug release rate. Besides, parts of CAM molecules attached onto MMT layers by hydrogen-bonding interactions may release more slowly, and thus can take part in slow release behavior of the drug delivery formulation.

To evaluate the type of drug release mechanism of the CAM-loaded formulations, the drug release data were fitted to the various kinetic models of Korsmeyer-Peppas (equation 12), Higuchi (equation 13), zero order (equation 14), and first order (equation 15), which are shown as follows:

(12) $${\text{M}}_{t}/M_{\infty }=kt^{n}$$

(13) $$M_{t}/M_{\infty }=K_{H}t^{1/2}$$

(14) $${\text{M}}_{t}/M_{\infty }=k_{0}t$$

(15) $$M_{t}/M_{\infty }=1-e^{-K_{1}t}$$

**Figure 7:**
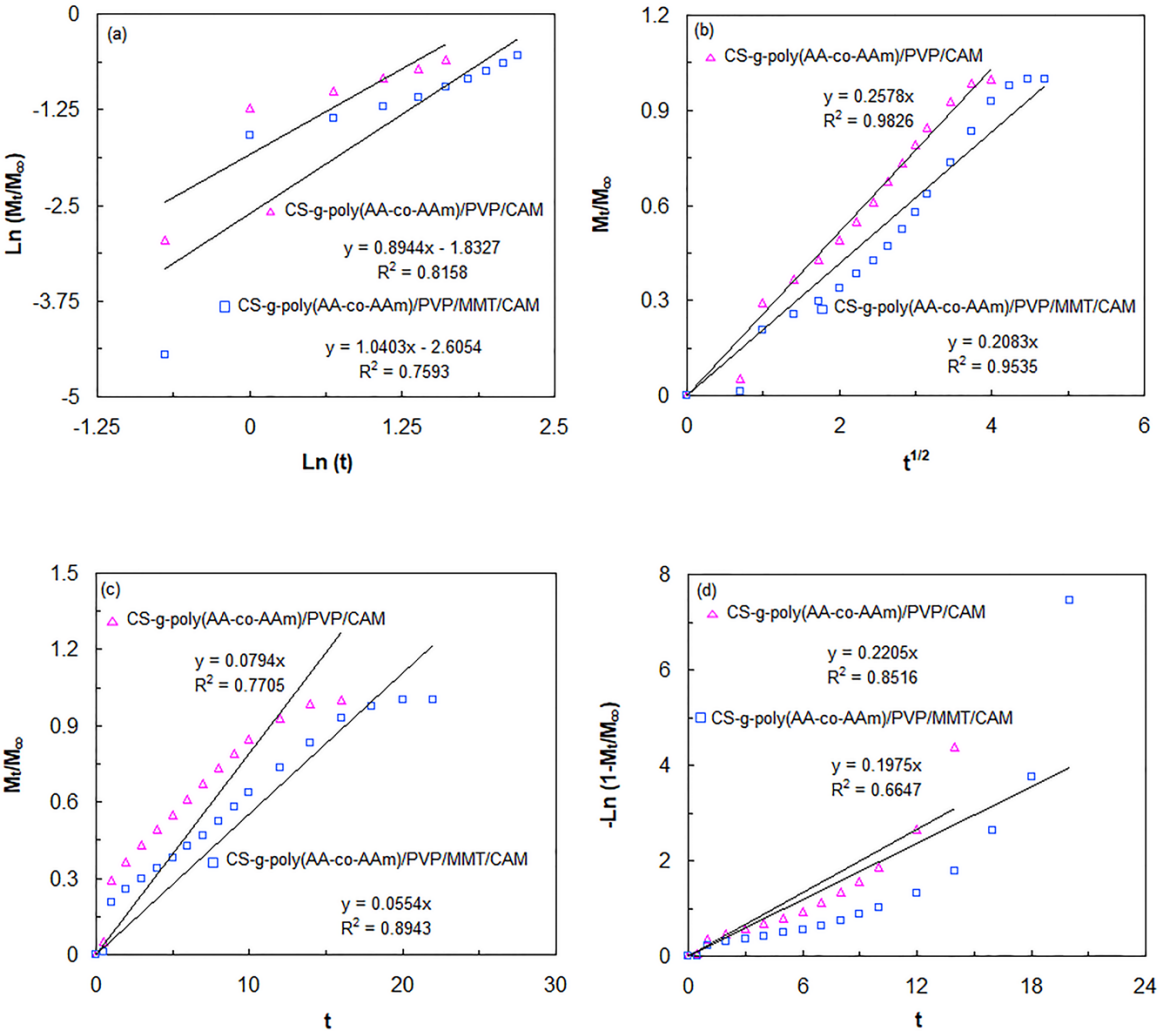
Plots of Ln (M_t_⁄M_∞_) versus Ln (t) (Korsmeyer-Peppas kinetic model) (a), plots of M_t_⁄M_∞_ versus t^1/2^ (Higuchi kinetic model) (b), plots of M_t_⁄M_∞_ versus t (zero order kinetic model) (c), and plots of –Ln (1- M_t_⁄M_∞_) versus t (first order kinetic model) (d) for CS-g-poly(AA-co-AAm)/PVP/CAM and CS-g-poly(AA-co-AAm)/PVP/MMT/CAM formulations.

Where M_t_/M_∞_ represents the fraction of the released drug and M_t_ and M_∞_ correspond to the cumulative amounts of released drug at time t and at infinite time (the maximum amount of the released drug). K, K_H_, H_0_, and K_1_ are corresponded to the rate constant, dissolution constant of Higuchi, kinetic dissolution constant, and first order release constant, respectively. *n* and *t* are also attributed to the diffusion exponent and release time, respectively. It is noteworthy that Korsmeyer-Peppas kinetic model is only applied at the initial stages of release (M_t_/M_∞_ < 0.6). The n ≤ 0.5 is assigned to the Fickian diffusion mechanism, while 0.5 < n <1 attributes to non-Fickian or anomalous diffusion mechanism type. When n = 1, case-II transport mechanism is occurred and when n >1, diffusion mechanism conforms to supercase–II transport behavior.^24,43,44^ The values of K and n in Korsmeyer-Peppas kinetic model were determined from the intercept and slope of the plotted lines (Figure 7a). Also, the slope of the plotted straight lines in Figure 7b (Higuchi kinetic model), Figure 7c (zero order kinetic model), and Figure 7d (first order kinetic model) was used to calculate the amounts of K_H_, H_0_, and K_1_ in the corresponding kinetic models, respectively. All these parameters and also correlation coefficients (R^2^) have been provided in Table 2. The best fit of each kinetic model was assessed by R^2^ values. According to Table 2, the best fit for drug release data of CS-g-poly(AA-co-AAm)/PVP/CAM and CS-g-poly(AA-co-AAm)/PVP/MMT/CAM formulations was found by Higuchi model. Therefore, the drug transport mechanism of both formulations follows from Fickian diffusion type.

**Table 2:** Parameters of Korsmeyer-Peppas, Higuchi, zero order, and first order drug release kinetic models for CS-g-poly(AA-co-AAm)/PVP/CAM and CS-g-poly(AA-co-AAm)/PVP/MMT/CAM formulations

**Kinetic model**	**Parameters**	**Formulations**	
		**CS-g-poly(AA-co-AAm)/PVP/CAM** **pH=1.2**	**CS-g-poly(AA-co-AAm)/PVP/MMT/CAM** **pH=1.2**
Korsmeyer-Peppas	R^2^	0.8158	0.7593
	n	0.8944	1.0403
	k × 10^2^	16.001	7.38
Higuchi	R^2^	0.9826	0.9535
	k_H_ × 10^2^	25.78	20.83
Zero-order	R^2^	0.7705	0.8943
	k_0_ × 10^2^	7.94	5.54
First-order	R^2^	0.8516	0.6647
	k_1_ × 10^2^	22.05	19.75

Superabsorbent nanocomposite developed in the present work possesses semi-IPN structure compared with that reported in literature.^45^ This structure enables it to absorb and preserve higher amount of aqueous solution within own network. According to the findings, it is evident that swelling capacity of semi-IPN CS-g-poly(AA-co-AAm)/PVP/MMT superabsorbent nanocomposite is higher than that of those described in the literature. The high water absorption capacity of superabsorbent nanocomposite not only can extend residence time of drug delivery formulation in stomach but also can significantly increase encapsulation efficiency (EE) of CAM. By comparing in vitro drug release results, it can be concluded that semi-IPN CS-g-poly(AA-co-AAm)/PVP/MMT/CAM formulation liberates CAM more slowly than that of formulation reported in literature, so that the time needed to release 50% of drug was 10 hours. At this condition, the effective life time of CAM in stomach for semi-IPN CS-g-poly(AA-co-AAm)/PVP/MMT/CAM formulation is prolonged, causing a substantial improvement in treatment of *H. pylori*-related infections. Incorporation of MMT into hydrogel network not only makes a considerable improvement in water absorption capacity and swelling rate of the semi-IPN superabsorbent nanocomposite but also amend its drug release rate. Also, utilization of MMT, as a low cost mineral filler, can significantly mitigate final production cost, and therefore practical use of the superabsorbent nanocomposite is feasible. Moreover, in the presence of MMT, due to its physical crosslinking effect, a stiff hydrogel framework with good gel strength is formed, which can withstand pressure caused by gastrointestinal movement. As a result, it can be expected that CS-g-poly(AA-co-AAm)/PVP/MMT/CAM formulation, due to the high effective life time of dug and sustained release character, can exert an efficient curative impact on the *H. pylori*-related gastric infection.

## Conclusion

A gastro-retentive drug delivery formulation of CAM based on semi-IPN CS-g-poly(AA-co-AAm)/PVP/MMT superabsorbent nanocomposite was prepared. FTIR results indicated that grafting reactions, PVP interpenetration through hydrogel network, nanocomposite formation, and also loading of CAM into hydrogel network have been done, successfully. According to the SEM images, in the presence of MMT, coarse surface morphology of hydrogel changes to a highly porous structure with interlinked channels. Swelling kinetic measurements indicated that superabsorbent nanocomposite (with 13 wt% MMT content) possesses greater equilibrium water absorption capacity (1568 g/g) and slower swelling rate compared with neat hydrogel (1270 g/g). According to the rheological studies, superabsorbent nanocomposite demonstrated stiff gel framework compared with the neat hydrogel, which results from physical crosslinking effect of the incorporated MMT. In vitro drug release assessments exhibited that CS-g-poly(AA-co-AAm)/PVP/MMT/CAM formulation can maintain CAM concentration in a simulated gastric medium (pH = 1.2) for prolonged period of time, indicating a sustain release character. These good characteristics revealed that the developed CS-g-poly(AA-co-AAm)/PVP/MMT/CAM formulation can be used as an effective drug delivery system to cure *H. pylori*-related infection. Currently, CS-based pH-sensitive formulations have significantly improved site-specific drug delivery efficiency at preclinical trials. However, several challenges have remained, which future researches should address them. It must be confirmed whether the developed formulation with targeted drug delivery for in vivo condition can cure *H. pylori*-related infection, efficiently. Also, the toxicological effects of the drug delivery formulations must be considered, precisely.

## Ethical Issues

Not applicable.

## Conflict of Interest

None declared.

## Acknowledgments

The authors would like to express their special thanks to drug applied research center staffs of Tabriz University of Medical Science for their kind cooperation and important help on this research work.

## References

[1] Boyer C, Boutevin G, Robin JJ, Boutevin B (2004). Study of the telomerization of dimethylaminoethyl methacrylate (DMAEMA) with mercaptoethanol Application to the synthesis of a new macromonomer. Polymer.

[2] Freichel OL, Lippold BC (2004). Artificially induced polymer particle erosion of oral hydrocolloid systems by the addition of insoluble cellulose fibres to fibre-free methylhydroxy ethylcellulose (MHEC). Eur J Pharm Biopharm.

[3] Ekici S, Saraydin D (2007). Interpenetrating polymeric network hydrogels for potential gastrointestinal drug release. Polym Int.

[4] Dergunov SA, Mun GA (2009). γ-irradiated chitosan-polyvinyl pyrrolidone hydrogels as pH-sensitive protein delivery system. Radiat Phys Chem.

[5] Peppas NA, Hilt JZ, Khademhosseini A, Langer R (2006). Hydrogels in biology and medicine: from molecular principles to bionanotechnology. Adv Mater.

[6] Bajpai AK, Shukla SK, Bhanu S, Kankane S (2008). Responsive polymers in controlled drug delivery. Prog Polym Sci.

[7] Myung D, Waters D, Wiseman M, Duhamel PE, Noolandi J, Ta CN (2008). Progress in the development of interpenetrating polymer network hydrogels. Polym Adv Technol.

[8] Bhattarai N, Gunn J, Zhang M (2010). Chitosan-based hydrogels for controlled, localized drug delivery. Adv Drug Deliv Rev.

[9] Zhang BY, He WD, Li WT, Li LY, Zhang KR, Zhang H (2010). Preparation of block-brush PEG-b-P(NIPAM-g-DMAEMA) and its dual stimulus-response. Polymer.

[10] Shin GH, Chung SK, Kim JT, Joung HJ, Park HJ (2013). Preparation of chitosan-coated nanoliposomes for improving the mucoadhesive property of curcumin using the ethanol injection method. J Agric Food Chem.

[11] Du H, Liu M, Yang X, Zhai G (2015). The design of pH-sensitive chitosan-based formulations for gastrointestinal delivery. Drug Discov Today.

[12] Gomez-Burgaz M, Garcia-Ochoa B, Torrado-Santiago S (2008). Chitosan-carboxymethylcellulose interpolymer complexes for gastric-specific delivery of clarithromycin. Int J Pharm.

[13] Verma A, Dubey J, Hegde RR, Rastogi V, Pandit JK (2016). Helicobacter pylori: past, current and future treatment strategies with gastroretentive drug delivery systems. J Drug Target.

[14] Goganian AM, Hamishehkar H, Arsalani N, Khiabani HK (2015). Microwave‐promoted synthesis of smart superporous hydrogel for the development of gastroretentive drug delivery system. Adv Polym Technol.

[15] Farshforoush P, Ghanbarzadeh S, Goganian AM, Hamishehkar H (2017). Novel metronidazole-loaded hydrogel as a gastroretentive drug delivery system. Iran Polym J.

[16] Chang CH, Lin YH, Yeh CL, Chen YC, Chiou SF, Hsu YM (2010). Nanoparticles incorporated in pH-sensitive hydrogels as amoxicillin delivery for eradication of Helicobacter pylori. Biomacromolecules.

[17] Ramteke S, Maheshwari RBU, Jain NK (2006). Clarithromycin based oral sustained release nanoparticulate drug delivery system. Indian J Pharm Sci.

[18] Nama M, Gonugunta CS, Reddy Veerareddy P (2008). Formulation and evaluation of gastroretentive dosage forms of Clarithromycin. AAPS PharmSciTech.

[19] Ozbas Z, Gurdag G (2015). Swelling kinetics, mechanical properties, and release characteristics of chitosan-based semi-IPN hydrogels. J Appl Polym Sci.

[20] Wang W, Wang Q, Wang A (2011). pH-responsive carboxymethylcellulose-g-poly (sodium acrylate)/polyvinylpyrrolidone semi-IPN hydrogels with enhanced responsive and swelling properties. Macromol Res.

[21] Yadav M, Rhee KY (2012). Superabsorbent nanocomposite (alginate-g-PAMPS/MMT): synthesis, characterization and swelling behavior. Carbohydr Polym.

[22] Lanthong P, Nuisin R, Kiatkamjornwong S (2006). Graft copolymerization, characterization, and degradation of cassava starch-g-acrylamide/itaconic acid superabsorbents. Carbohydrate Polymers.

[23] Ibrahim NA, Yunus WM, Abu-Ilaiwi FA, Rahman MZ, Bin Ahmad M, Dahlan KZM (2003). Graft copolymerization of methyl methacrylate onto oil palm empty fruit bunch fiber using H2O2/Fe2+ as an initiator. J Appl Polym Sci.

[24] Farshi Azhar F, Olad A (2014). A study on sustained release formulations for oral delivery of 5-fluorouracil based on alginate–chitosan/montmorillonite nanocomposite systems. Appl Clay Sci.

[25] Uthirakumar P, Nahm KS, Hahn YB, Lee YS (2004). Preparation of polystyrene/montmorillonite nanocomposites using a new radical initiator-montmorillonite hybrid via in situ intercalative polymerization. Eur Polym J.

[26] Zhang J, Wang L, Wang A (2007). Preparation and properties of chitosan-g-poly (acrylic acid)/montmorillonite superabsorbent nanocomposite via in situ intercalative polymerization. Ind Eng Chem Res.

[27] Patel HA, Somani RS, Bajaj HC, Jasra RV (2007). Preparation and characterization of phosphonium montmorillonite with enhanced thermal stability. Appl Clay Sci.

[28] Wang W, Wang A (2010). Synthesis and swelling properties of pH-sensitive semi-IPN superabsorbent hydrogels based on sodium alginate-g-poly (sodium acrylate) and polyvinylpyrrolidone. Carbohydr Polym.

[29] Lu S, Liu M, Ni B, Gao C (2010). A novel pH‐and thermo‐sensitive PVP/CMC semi‐IPN hydrogel: Swelling, phase behavior, and drug release study. J Polym Sci B Polym Phys.

[30] Wang X, Zhou Z, Guo X, He Q, Hao C, Ge C (2016). Ultrasonic-assisted synthesis of sodium lignosulfonate-grafted poly (acrylic acid-co-poly (vinyl pyrrolidone)) hydrogel for drug delivery. RSC Adv.

[31] Vaghani SS, Patel MM (2011). pH-sensitive hydrogels based on semi-interpenetrating network (semi-IPN) of chitosan and polyvinyl pyrrolidone for clarithromycin release. Drug Dev Ind Pharm.

[32] Bagheri Marandi G, Mahdavinia GR, Ghafary S (2011). Collagen-g-poly (Sodium Acrylate-co-Acrylamide)/sodium montmorillonite superabsorbent nanocomposites: synthesis and swelling behavior. J Polym Res.

[33] Zhang J, Wang Q, Wang A (2007). Synthesis and characterization of chitosan-g-poly (acrylic acid)/attapulgite superabsorbent composites. Carbohydr Polym.

[34] Bao Y, Ma J, Li N (2011). Synthesis and swelling behaviors of sodium carboxymethyl cellulose-g-poly (AA-co-AM-co-AMPS)/MMT superabsorbent hydrogel. Carbohydr Polym.

[35] Zhang J, Wang A (2007). Study on superabsorbent composites IX: Synthesis, characterization and swelling behaviors of polyacrylamide/clay composites based on various clays. React Funct Polym.

[36] Qiu L, Chen W, Qu B (2006). Morphology and thermal stabilization mechanism of LLDPE/MMT and LLDPE/LDH nanocomposites. Polymer.

[37] Taleb MFA (2008). Radiation synthesis of polyampholytic and reversible pH-responsive hydrogel and its application as drug delivery system. Polym Bull.

[38] Mahdavinia GR, Pourjavadi A, Hosseinzadeh H, Zohuriaan MJ (2004). Modified chitosan 4 Superabsorbent hydrogels from poly (acrylic acid-co-acrylamide) grafted chitosan with salt-and pH-responsiveness properties. Eur Polym J.

[39] Rashidzadeh A, Olad A, Reyhanitabar A (2015). Hydrogel/clinoptilolite nanocomposite-coated fertilizer: swelling, water-retention and slow-release fertilizer properties. Polym Bull.

[40] Kasgoz H, Durmus A (2008). Dye removal by a novel hydrogel-clay nanocomposite with enhanced swelling properties. Polym Adv Technol.

[41] Geever LM, Lyons JG, Higginbotham CL (2011). Photopolymerisation and characterisation of negative temperature sensitive hydrogels based on N, N-diethylacrylamide. J Mater Sci.

[42] Fathi M, Farajollahi AR, Entezami AA (2013). Synthesis of fast response crosslinked PVA-g-NIPAAm nanohydrogels by very low radiation dose in dilute aqueous solution. Radiat Phys Chem.

[43] Dragan ES, Cocarta AI, Gierszewska M (2016). Designing novel macroporous composite hydrogels based on methacrylic acid copolymers and chitosan and in vitro assessment of lysozyme controlled delivery. Colloids Surf B Biointerfaces.

[44] Kumar P, Ganure AL, Subudhi BB, Shukla S (2015). Preparation and characterization of pH-sensitive methyl methacrylate-g-starch/hydroxypropylated starch hydrogels: in vitro and in vivo study on release of esomeprazole magnesium. Drug Deliv Transl Res.

[45] Kumar GA, Wadood SA, Datta MS, Ramchand D (2010). Interpenetrating polymeric network hydrogel for stomach-specific drug delivery of clarithromycin: Preparation and evaluation. Asian J Pharm.

